# Are working memory and glutamate concentrations involved in early‐life stress and severity of psychosis?

**DOI:** 10.1002/brb3.1616

**Published:** 2020-05-09

**Authors:** Mark Corcoran, Emma L. Hawkins, Denis O'Hora, Heather C. Whalley, Jeremy Hall, Stephen M. Lawrie, Maria R. Dauvermann

**Affiliations:** ^1^ School of Psychology National University of Ireland Galway Galway Ireland; ^2^ Division of Psychiatry University of Edinburgh Edinburgh UK; ^3^ Neuroscience and Mental Health Research Institute Cardiff University School of Medicine Cardiff UK; ^4^ McGovern Institute for Brain Research Massachusetts Institute of Technology Cambridge MA USA; ^5^ Department of Psychiatry University of Cambridge Cambridge UK

**Keywords:** early‐life stress, glutamate, left dorsolateral prefrontal cortex, psychosis, working memory

## Abstract

**Objective:**

Occurrences of early‐life stress (ELS) are associated with the severity of psychotic symptoms and working memory (WM) deficits in patients with psychosis (PSY). This study investigated potential mediation roles of WM behavioral performance and glutamate concentrations in prefrontal brain regions on the association between ELS and psychotic symptom severity in PSY.

**Method:**

Forty‐seven patients with PSY (established schizophrenia, *n* = 30; bipolar disorder, *n* = 17) completed measures of psychotic symptom severity. In addition, data on ELS and WM performance were collected in both patients with PSY and healthy controls (HC; *n* = 41). Resting‐state glutamate concentrations in the bilateral dorsolateral prefrontal cortex (DLPFC) and anterior cingulate cortex (ACC) were also assessed with proton magnetic resonance spectroscopy for both PSY and HC groups. *t* tests, analyses of variance, and regression analyses were utilized.

**Results:**

Participants with PSY reported significantly more ELS occurrences and showed poorer WM performance than HC. Furthermore, individuals with PSY displayed lower glutamate concentrations in the left DLPFC than HC. Neither ELS nor WM performance were predictive of severity of psychotic symptoms in participants with PSY. However, we found a significant negative correlation between glutamate concentrations in the left DLPFC and ELS occurrence in HC only.

**Conclusion:**

In individuals with PSY, the current study found no evidence that the association between ELS and psychotic symptoms is mediated by WM performance or prefrontal glutamate concentrations. In HC, the association between ELS experience and glutamate concentrations may indicate a neurometabolite effect of ELS that is independent of an illness effect in psychosis.

## Graphical abstract

This study investigated mediation roles of working memory performance and glutamate concentrations in prefrontal brain regions on the association between the occurrence of early‐life stress and symptom severity in individuals with schizophrenia and bipolar disorder. Our findings of reduced glutamate concentrations in the left dorsolateral prefrontal cortex and poorer working memory performance in patients with bipolar disorder, but not schizophrenia, may be useful for improved diagnosis in psychosis and treatment.

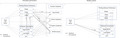

## SIGNIFICANT OUTCOMES

Our findings of reduced glutamate concentrations in the left dorsolateral prefrontal cortex and poorer working memory performance in patients with psychosis (including both schizophrenia and bipolar disorder) provide evidence for illness‐related changes.

The significant relationship between early‐life stress experience and glutamate concentrations in the left dorsolateral prefrontal cortex in healthy controls indicates preliminary evidence for general neurobiological effect of ELS.

## LIMITATIONS

The early‐life stress measure used in this study does not directly ask participants about abusive and neglectful experiences during childhood although it is known that such experiences are highly prevalent in individuals with a psychotic disorder. This may account for the lack of a relationship between early‐life stress occurrences and psychotic symptom severity in our findings. However, it is also known that other environmental risk factors are implicated in the onset and severity of psychosis.

Due to the relatively small sample, our analyses were not sensitive to weaker associations among early‐life stress occurrences, working memory performance, glutamate concentrations, and psychotic symptom severity.

## 1 INTRODUCTION

Psychotic disorders are debilitating conditions that are primarily characterized by positive symptoms (including delusions, hallucinations, and disorganized thoughts), negative symptoms (such as avolition, alogia, or apathy), and cognitive deficits (such as social cognition and working memory impairments). The latter can occur prior to the diagnosis of a psychotic disorder and can worsen with illness progression (Fusar‐Poli et al., 2010; MacDonald & Schulz, 2009). Cognitive deficits are widely considered to be core symptoms of schizophrenia (SZ; Gold, 2004) and bipolar disorder (BD; Bortolato, Miskowiak, Köhler, Vieta, & Carvalho, 2015; Martinez‐Aran & Vieta, 2015) and are also associated with reductions in working memory performance. Working memory deficits are one of the main neurocognitive impairments found in subjects with first‐episode psychosis (Kim et al., 2011; Seidman, 2010) and in patients at the established stage (Genevsky, Garrett, Alexander, & Vinogradov, 2010). Deficits in working memory are important given the role of working memory in information retention and manipulation in order to perform abilities such as speech and multitasking (Vöhringer et al., 2013) and involve the recruitment of the dorsolateral prefrontal cortex (DLPFC; Glahn et al., 2005; Jalbrzikowski et al., 2018; Roberts, Libby, Inhoff, & Ranganath, 2018). Furthermore, working memory deficits are associated with impaired daily functioning in patients with a psychotic disorder (Vesterager et al., 2012).

Epidemiological and clinical studies have also consistently reported the impact of stress‐related environmental risk factors on the severity of psychotic symptoms in patients with SZ and BD, such as early‐life stress (ELS) which includes physical abuse, physical neglect, emotional abuse, emotional neglect, sexual abuse and household dysfunction (Bechdolf et al., 2010; Day et al., 1987; Gershon, Johnson, & Miller, 2013; Holtzman et al., 2013; Lataster, Myin‐Germeys, Lieb, Wittchen, & Os, 2012; Mayo et al., 2017; Os, Kenis, & Rutten, 2010; Shannon et al., 2011; Thompson et al., 2009; Üçok et al., 2015; Varese et al., 2012; Walder, Faraone, Glatt, Tsuang, & Seidman, 2014) among cannabis use (Murray et al., 2017) and urbanicity (McGrath et al., 2004; Newbury et al., 2018). In addition, recent evidence suggests that the occurrence of ELS also impacts on neurocognitive function, such as working memory performance in both individuals with PSY and HC (Dauvermann & Donohoe, 2019a; Goodman, Freeman, & Chalmers, 2019; Vargas et al., 2019) as well as brain activation and connectivity in HC (Philip et al., 2016; Philip et al., 2013). However, little is known about the underlying mechanism between the environmental risk factor of ELS and working memory function in individuals with PSY.

The glutamate hypothesis of schizophrenia (Coyle, 2012) and the *N*‐methyl‐D‐aspartate receptor (NMDA‐R) hypofunction model (Coyle, 1996) posit that aberrant glutamate systems (Krystal et al., 1994) may be implicated in the pathophysiology of schizophrenia and psychosis. Proton magnetic resonance spectroscopy (^1^H‐MRS) studies examined both the glutamate hypothesis of schizophrenia and the NMDA‐r hypofunction model by measuring resting‐state glutamate levels in prefrontal brain regions in individuals with SZ and BP. Such studies reported inconsistent findings of glutamate levels in the dorsolateral prefrontal cortex (DLPFC) in patients with SZ (Poels et al., 2014) with studies reporting (a) increased glutamate levels (Olbrich et al., 2008; Rüsch et al., 2008), (b) decreased glutamate levels, (Ćurčić‐Blake et al., 2017) or (c) no difference in glutamate levels (Yoo et al., 2009) in the DLPFC compared to healthy controls (HC). For glutamate levels in the ACC in patients with SZ, there are further inconsistent findings with the majority of studies demonstrating no difference in concentrations of glutamate or glutamate‐related metabolites, such as glutamine or glutamate and glutamine combined (Glx) concentrations between chronic patients with SZ and HC (Bustillo et al., 2011; Kraguljac, Reid, White, Hollander, & Lahti, 2012; Reid et al., 2010; Rowland et al., 2013; Shirayama et al., 2010). However, a small number of studies also reported decreased concentrations of glutamate between patients with SZ and HC (Tayoshi et al., 2009; Théberge et al., 2003, 2004). The pattern of findings in patients with BD differs from patients with SZ, where individuals with BD consistently showed higher Glx levels in the DLPFC and ACC when compared to HC (Öngür et al., 2008; Soeiro‐de‐Souza et al., 2016). Focusing on working memory deficits, it has been proposed that altered glutamate regulation is one of the main neurobiological pathways underlying working memory impairments in psychosis based on both preclinical and clinical evidence (Dauvermann, Lee, & Dawson, 2017; Dauvermann et al., 2014; Javitt, 2007). Further support for the role of glutamate during working memory in individuals with PSY comes from a recent study using functional ^1^H‐MRS (Jelen et al., 2019).

In the last few years, emerging evidence proposes a role of stress, in particular social stress, in aberrant glutamatergic transmission in the PFC in rodents (Schiavone et al., 2020; Zhang, Hernández, Vázquez‐Juárez, Chay, & Barrio, 2016), which has also been in shown in HC who have experienced ELS (Duncan et al., 2015). Importantly, it has been shown that stress, in particular chronic social stress, resulted in both deficits in working memory and glutamatergic dysregulation in the PFC in rodents (Onaolapo et al., 2019; Shao et al., 2015; Yuen et al., 2011). However, to date no study has examined the association between ELS experience and severity of psychotic symptoms in individuals with PSY when considering glutamate concentrations as a potential neurobiological mechanism, including working memory function (Aas et al., 2012; Janssen et al., 2004).

### 1.1 Aims of the study

In this study, we investigated whether there is a relationship between the experience of ELS and severity of psychotic symptoms in PSY. In addition, we examined whether working memory performance and glutamate concentrations during resting state in the bilateral DLPFC and the ACC as measured with ^1^H‐MRS mediate this relationship between the ELS experience and severity of psychotic symptoms.

## 2 MATERIAL AND METHODS

### 2.1 Participants

Forty‐seven participants with psychosis (SZ = 30, BD = 17) and 41 HC were recruited for the study. PSY and HC were recruited from the Royal Edinburgh Hospital, associated hospitals, and the Scottish Mental Health Research Register (http://www.smhrn.org.uk/). Diagnosis of SZ and BD was based on interview using the Structured Clinical Interview for DSM‐IV (First, Spitzer, Gibbon & Williams, 2002). Inclusion criteria included (a) diagnosis of established SZ or BD, and (b) no acute psychotic symptoms at the time of the scan. Exclusion criteria included (a) history of any major psychiatric illness other than SZ or BD, (b) history of severe brain injury, (c) history of a neurological disorder, and (d) dependency or harmful use of alcohol or drugs during the last 12 months. Also, HC were excluded if they had a family history of SZ or BD. All participants provided written informed consent. The study was approved by the local Research Ethics Committee (09/MRE00/81).

### 2.2 Measures

#### 2.2.1 Demographic measures

Participants provided information regarding their age, sex, and education status. Participants were also asked regarding medication use (antipsychotic medication, mood stabilizers, and antidepressant medication).

Smoking status has not been recorded.

#### 2.2.2 Clinical measures

The Positive and Negative Syndrome Scale (PANSS; Kay, Fiszbein, & Opler, 1987) is a 30‐item standardized clinical interview to rate the presence and severity of positive and negative symptoms in PSY. The measure consists of the following scales: positive symptoms, negative symptoms, general symptoms, and total score. Items were rescored to 0 (“Absent”) to 6 (“Extreme”) for symptoms during the past week with higher scores indicating greater severity. Internal reliability for the three subscales of positive symptoms, negative symptoms, and general symptoms has been reported as adequate (Cronbach's *α* .73–.83; Kay et al., 1987). Previous studies have indicated adequate validity of the PANSS (Kay et al., 1987). Symptom rating took place within 1 week of the ^1^H‐MRS acquisition.

#### 2.2.3 Environmental questionnaires

The Childhood Life Events Questionnaire (CLEQ; http://bdrn.org/) is a 13‐item verbally administered measure of ELS shared by the Bipolar Disorder Research Network (BDRN [Upthegrove et al., 2015]). All participants were asked whether they had experienced any or all of a list of 12 adverse events prior to the age of 16 years as previously reported (Barker et al., 2016; Neilson et al., 2017). This list includes death of a parent, death of a sibling, death of a friend, parental separation, parental divorce, admission to hospital, hospitalization of parent, visible deformity, teenage pregnancy or fatherhood, imprisoned parent and suspension from school. Responses ranged from 0 (“No”) to 1 (“Yes”). While no questions regarding childhood abuse or neglect were asked, participants were given the opportunity to provide this information by the final item which is an open‐ended question: “Are there any other significant life events you experienced as a child that are not mentioned above?”

### 2.3 Working memory data

All participants performed the verbal “2‐back” task. They were presented with a sequence of single capital letters. The experimental block design consisted of (a) the baseline or “0‐back” condition; (b) the “1‐back” condition; and (c) the “2‐back” condition. Full experimental details are presented in the Appendix S1.

### 2.4 Brain acquisition and analysis

#### 2.4.1 Structural MRI acquisition and analysis

Brain scans were collected using a 3 T Siemens Verio MRI scanner using a manufacturer‐supplied 12‐element matrix head coil at the Clinical Research Imaging Centre (CRIC), the Queen's Medical Research Institute, Edinburgh, UK. After a sagittal localizer, T1‐weighted magnetization‐prepared rapid‐acquisition gradient echo (MPRAGE) MR images were obtained using TR = 2,300 ms, TE = 2.98 ms, and TI = 900 ms, (flip angle = 9, FOV = 256 mm × 256 mm) with an isotropic voxel resolution of 1 mm, parallel to AC‐PC plane.

#### 2.4.2 Proton magnetic resonance spectroscopy data acquisition and analysis

##### Magnetic resonance spectroscopy data acquisition

We acquired MRS point‐resolved selective spectroscopy (PRESS) spectra with voxel placements in the ACC, right DLPFC, and left DLPFC. The voxels were shimmed using the Siemens advance mode. We acquired a water unsuppressed spectra with 16 averages and a water suppressed spectra with 128 averages. The TE was set to 80ms and the TR was set to 3,000 ms. The phase cycling was set to the Siemens 16 EXOR‐cycle mode and the bandwidth was 2,500 Hz with oversampling enabled. The voxels in the DLPFC measure 20 × 20 × 20 mm (8 cm^3^) and in the ACC measures 30 × 20 × 15 mm (9 cm^3^).

**FIGURE 1:**
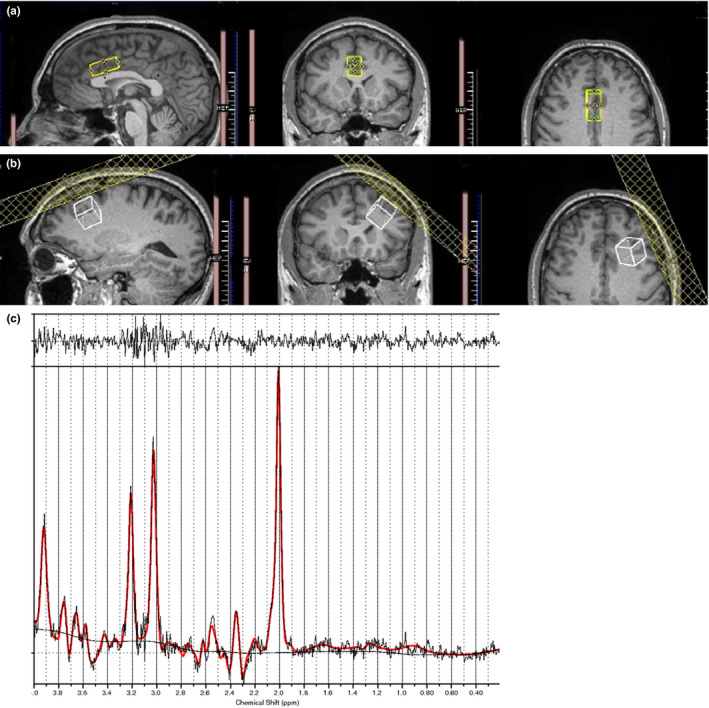
Voxel placement and example of magnetic resonance spectroscopy spectrum. (a) Voxel placement for the anterior cingulate cortex. (b) Voxel placement for the dorsolateral prefrontal cortex. (c) Example for glutamate spectrum

This followed a standardized protocol, with navigation steps to determine the coronal slice for placement. Cortical feature identification was used to designate the voxel center, followed by rotations of the voxel in the transverse and sagittal views to obtain the final placement. The location of the ACC voxel is shown in Figure 1a and the placement of the left DLPFC voxel is shown in Figure 1b. Details for the voxel placement are presented in the Appendix S1 and in a previous paper (Thomson et al., 2016). An example of a spectrum acquired in the dACC in a HC (Figure 1c).

##### Metabolite measurement

Spectral metabolite quantification was performed in LCModel version 6.2 (http://www.s‐provencher.com/pages/lcmodel.shtml) using a gamma_press_te80_123mhz basis set provided at http://s‐provencher‐com for use with the Siemens 3T Verio scanner. LCModel obtains maximum‐likelihood estimates of metabolite concentrations and their uncertainties (Cramer–Rao lower bounds; CRLB). The quality of the spectra obtained and the specificity of the measurement of a given metabolite were evaluated using the CRLB measure of uncertainty. This is a measure of the specificity of the peak in the spectrum associated with a given metabolite. Only metabolite measurements that were associated with a fitting error (CRLB) of <15% were included in the analysis.

The raw spectra were read into the graphical user interface for LCModel and processed through LC Model with eddy current correction enabled and internal water reference was used to give the metabolite in institutional units. For each voxel the metabolite values derived from LCModel were corrected for voxel cerebrospinal fluid (CSF) content as per the equation:

(1) $${\text{Met}}_{\text{CI}}={\text{Met}}_{I}\times \left(\frac{1}{1-F_{\text{CSF}}}\right)$$

where Met_CI_ is the metabolite in institutional units and corrected for partial volume effects, Met_I_ is the internal water scaled metabolite value given by LC model, and F_CSF_ is the fractional CSF occupancy of the voxel. The fractional CSF volume was determined from the segmentation of the T_1_‐weighted scan and the voxel placement and rotation noted at scan time. The T1‐weighted images were segmented into gray matter, white matter, and CSF maps using SPM8 (Statistical Parametric Mapping; http://fil.ion.ucl.ac.uk/spm/). The CSF volume in each voxel was extracted using a c‐script that sampled the SPM segmentation maps at the native space location noted for each voxel at time of scan.

### 2.5 Statistical analysis

#### 2.5.1 Demographic data

Frequencies, means, and standard deviations were calculated for sex, age, and level of education in Statistical Package for Social Sciences version 25 (SPSS; IBM, 2017). Smoking status was not recorded.

#### 2.5.2 Clinical and environmental data

A series of *t* tests were conducted to examine differences between groups on measures, such as PANSS total scores, PANSS positive symptoms, PANSS negative symptoms, PANSS general scores, ELS data, and glutamate concentrations (separately in the right DLPFC, left DLPFC, and ACC). In following analyses, a multivariate ANOVA (MANOVA) was conducted to examine group differences in glutamate concentrations in the bilateral DLPFC and ACC. Multiple linear regressions assessed whether ELS occurrences, WM performance, or glutamate metabolite concentrations (separately in the bilateral DLPFC and ACC) predicted psychotic symptom severity by introducing these predictors systematically across blocks. Statistical significance was set at a *p* value below .05. Pearson's product‐moment correlations were computed to examine the relationships between ELS occurrence, WM behavioral performance, glutamate metabolite concentrations, and psychotic symptom severity. *p* values were adjusted for multiple comparison using the Hochberg method in R Studio (RStudio Team, 2018).

#### 2.5.3 Working memory data

Behavioral performance was calculated using the sensitivity index (*d*′) (Equation 1; Macmillan, Creelman, & Creelman, 2004). Behavioral performance on the 0‐back, 1‐back, and 2‐back conditions between the groups was compared in SPSS using independent *t* tests and ANOVAs.

(2) $$\begin{array}{c} d^{\prime }=z\left(\text{Hits}\right)-z\left(\text{False}\;\text{Alarms}\right)\\ z=\text{statistical}\;Z\;\text{value} \end{array}$$

#### 2.5.4 Glutamate concentrations

Analysis of covariance (ANCOVA) was performed to examine the hypothesized differences in glutamate concentrations in each region between the PSY and HC groups. Age and sex were entered in as covariates for all comparisons.

## 3 RESULTS

### 3.1 Demographic, clinical, and working memory data

Demographic and clinical data are presented in Table 1. Ages ranged from 18 to 67 years (*M = *39.5, *SD* = 12.99) with an average of 90 months of illness duration. Most participants (55%) completed postsecondary education. The majority of participants who took part in the study were male (66.3%). Demographic and clinical data for SZ and BD subgroups are presented in the Appendix S1 (Table S1).

**TABLE 1:** Demographic and clinical details

	HC	PSY	Test	*p*
*N*	41	47		
Age Mean (*SD*)	38.29 (14.44)	40.62 (11.63)	*t*	.406
Sex (M:F)	23:18	33:14	*χ* ^2^	.170
Education (1:2:3)^a^	10:2:24	8:7:24	*χ* ^2^	.112
PANSS total^b^ Mean (*SD*)	–	23.28 (17.87)	–	–
PANSS positive^b^ Mean (*SD*)	–	5.30 (5.00)	–	–
PANSS negative^b^ Mean (*SD*)	–	6.38 (7.11)	–	–
PANSS general^b^ Mean (*SD*)	–	11.60 (8.60)	–	–
CLEQ^c^ Mean (*SD*)	1.23 (1.48)	2.49 (1.90)	*t*	.001*

Abbreviations: CLEQ, Childhood Life Events Questionnaire; HC, healthy controls; PANSS, Positive and Negative Symptom Scale.

^a^ 0, compulsory; 1, more than compulsory; 2, postsecondary.

^b^ Rescaled total PANSS scores.

^c^ Rescaled CLEQ scores.

* *p* < .001.

#### 3.1.1 Clinical data

##### Psychotic symptom severity

Positive and Negative Syndrome Scale severity in PSY was assessed with comparable severity (Table 1). In addition, independent *t* tests revealed a significant subgroup difference in PANSS positive symptoms (*t*_(45)_ = 2.36, *p* = .046, *d* = 0.76), with the SZ group scoring higher than the BD group. There were no significant group differences in PANSS total scores (*t*_(45)_ = 0.945, *p* = .220, *d* = 0.29), PANSS negative symptoms (*t*_(45)_ = 1.26, *p* = .160, *d* = 0.42), or PANSS general symptoms (*t*_(45)_ = −0.38, *p* = .543, *d* = 0.66; Table S1).

#### 3.1.2 Early‐life stress

The following reported experiencing no (*n*; HC = 15; PSY = 8), one (HC = 11; PSY = 6), two (HC = 8; PSY = 6), three (HC = 2; PSY = 10), or four or more (HC = 3, PSY = 11) occurrences of ELS. Regarding childhood trauma (i.e., abuse and/or neglect), 2.4% of HC reported such experiences, compared to 12.2% of PSY participants (SZ = 13.3%, BD = 11.7%). An independent *t* test (PSY vs. HC) revealed a significant group difference in ELS scores (*t*_(78)_ = 3.29, *p* = .001, *d* = 0.74) with PSY reporting greater occurrence (*M* = 2.49, *SD* = 1.90) than HC (*M* = 1.23, *SD* = 1.48). A one‐way ANOVA (SZ vs. BD vs. HC) revealed a significant subgroup difference in ELS scores (*F*_(2, 79)_ = 6.729, *p* = .002, *η* ^2^ = .149). Post hoc analyses revealed a significant group difference between the HC and the SZ groups (*p* = .001) in ELS scores, with the SZ group scoring higher (see Table S1). There was no significant group difference between the BD and HC groups (*p* = .267) and the BD and SZ groups (*p* = .272) on ELS scores.

#### 3.1.3 Working memory performance

To examine group differences in behavioral performance on the N‐back task, a series of *t* tests were conducted (HC vs. PSY). There was no significant difference on the 0‐back performance (*t*_(74)_ = 1.622, *p* = .109, *d* = 0.372). For both the 1‐back and 2‐back conditions, PSY performed less accurately than HC. Regarding 1‐back performance, there was a significant group difference (*t*_(74)_ = 3.212, *p* = .002, *d* = 0.734), with the PSY group (*M* = −0.60, *SD = *1.87) scoring lower than the HC group (*M = *0.57, *SD = *1.26). Regarding 2‐back performance, a significant group difference was also observed (*t*_(74)_ = 2.60, *p* = .011, *d* = 0.594) with the PSY group (*M* = −0.47, *SD = *1.70) performing worse than the HC group (*M = *0.45, *SD = *1.38).

To examine any differences in N‐back performance between subgroups, a series of one‐way ANOVAs (HC vs. SZ vs. BD) were conducted. Regarding behavioral performance on the 0‐back task, we did not find any significant differences between the three groups (*F*_(2,76)_ = 1.31, *p* = .277, *η* ^2^ = .035). Regarding the 1‐back task, a significant difference was observed (*F*_(2,76)_ = 5.121, *p* = .008, *η* ^2^ = .123). Post hoc analyses revealed that the SZ group performed significantly poorer (*p* = .012) than the HC group. There was no significant difference between the SZ and BD groups (*p* = .970) or the BD and HC groups (*p* = .092). On the 2‐back task, a significant group difference was also found (*F*_(2,76)_ = 3.357, *p* = .040, *η* ^2^ = .084). Post hoc analyses revealed that the SZ group scored significantly lower (*p* = .049) than the HC group. There were no significant differences between the SZ and BD groups (*p* = .961) or between the BD and HC groups (*p* = .228).

### 3.2 Glutamate concentrations

The number of participants who met the criterion of data quality is presented in Table 2. A series of one‐way ANCOVAs (PSY vs. HC) were conducted to examine whether glutamate concentrations in the three brain areas varied according to group, while controlling for age and sex. The results of the testing found a significant difference in glutamate concentration in the left DLPFC between groups (*F*_(1, 63)_ = 5.742, *p* = .020, partial *η* ^2^ = .084), with the PSY group reporting lower concentrations of glutamate than the HC group (see Table 2). There were no significant differences between the PSY and HC groups with regards to glutamate concentration in the right DLPFC (*p* = .324) or ACC (*p* = .971). Extension of the inclusion criteria (glutamate standard deviation <30%) resulted in no significant difference in glutamate concentrations in the bilateral DLPFC or ACC between the PSY and HC groups (*p* > .05). These analyses were repeated for subgroups (SZ vs. BD vs. HC). No significant difference was found for the right or left DLPFC or ACC (see Appendix S1; Table S2).

**TABLE 2:** Glutamate concentrations in institutional units (IU)^a,^, ^b^

	HC	PSY	Test	*p*
R DLPFC	8.64 (2.11) *n* = 33	8.04 (1.46) *n* = 29	*F*	.324
L DLPFC	8.20 (1.36) *n* = 34	7.41 (1.37) *n* = 33	*F*	.020*
ACC	7.79 (1.71) *n* = 28	7.17 (1.87) *n* = 35	*F*	.971

Abbreviations: ACC, anterior cingulate cortex; HC, healthy controls; L DLPFC, left dorsolateral prefrontal cortex; PSY, individuals with psychosis; R DLPFC, right dorsolateral prefrontal cortex.

^a^ Age and sex were entered as covariates.

^b^ Participants with glutamate concentration standard deviation exceeding 15% were excluded from this table.

* *p* < .05.

### 3.3 Correlational analyses

Pearson's bivariate correlations were used to identify associations between severity of clinical symptoms, ELS occurrences, glutamate concentrations (right DLPFC, left DLPFC and ACC), and WM performance in a series of analyses. First, severity of clinical symptoms and occurrence of ELS in PSY only were tested (Table 3). Second, correlations between ELS and glutamate concentrations in both HC and PSY were run separately of each of the three brain regions (right DLPFC, left DLPFC and ACC; Table 4). Finally, relationships between ELS experience and WM performance were examined for both groups (Table 5). *p* values were adjusted for multiple comparisons using the Hochberg method. The *α* level was set at .05.

**TABLE 3:** Pearson's correlation coefficients for severity of clinical symptoms and occurrence of early‐life stress for individuals with psychosis

	PSY
*N*	41

PANSS scale	CLEQ scale	
	*r*	*p*
Total	.009	.996
Positive symptoms	.011	.996
Negative symptoms	.052	.996
General symptoms	−.030	.996

Abbreviations: PANSS, Positive and Negative Symptom Scale; PSY, individuals with psychosis.

* denotes significant at *p* < .05.

**TABLE 4:** Pearson's correlation coefficients for glutamate concentrations and occurrence of early‐life stress for healthy controls and individuals with psychosis

Brain region	HC	PSY
R DLPFC		
*r*	−.088	−.045
*p*	.703	.827
*n*	30	26
L DLPFC		
*r*	−.449	.163
*p*	.029^*^	.577
*n*	31	31
ACC		
*r*	.131	.306
*p*	.514	.089
*n*	27	32

Abbreviations: ACC, anterior cingulate cortex; HC, healthy controls; L DLPFC, left dorsolateral prefrontal cortex; PSY, individuals with psychosis; R DLPFC, right dorsolateral prefrontal cortex.

* denotes significant at*p* < .05.

**TABLE 5:** Pearson's correlation coefficients for behavioral performance during working memory and occurrence of early‐life stress for healthy controls and individuals with psychosis

	HC	PSY
*N*	37	32

N‐back	*r*	*p*	*r*	*p*
0‐back	−.343	.114	.025	.891
1‐back	−.050	.888	−.052	.888
2‐back	−.052	.921	.122	.921

Abbreviations: HC, healthy controls; PSY, individuals with psychosis.

There were no significant correlations between the occurrence of ELS and severity of psychotic symptoms in the PSY group (all *p* > .05; Table 3). Regarding glutamate levels, a significant negative correlation between the occurrence of ELS and glutamate concentration in the left DLPFC (*r* = −.449, *p* = .029; Table 4) was observed in the HC group only. In the PSY group, there were no significant correlations between the occurrence of ELS and concentrations of glutamate in the right DLPFC, left DLPFC, or ACC (all *p *> .05). We also did not observe any significant correlations between the occurrence of ELS and working memory performance in the HC or PSY group (all *p *> .05; Table 5).

### 3.4 Predictors of severity of psychotic symptoms

For the PSY group, occurrences of ELS, working memory performance and glutamate concentrations were systematically added across blocks in four multiple linear regressions predicting severity of psychotic symptoms (PANSS total score, PANSS positive symptoms, PANSS negative symptoms and PANSS general score). This process was repeated for glutamate concentrations in each of the three brain regions (right DLPFC, left DLPFC, and ACC). No model was predictive of psychotic symptoms (all *p *> .05).

In addition, we repeated these analyses to predict the relationship between ELS, working memory, and psychotic symptoms for the BD and SZ groups separately. No model significantly predicted symptoms in the SZ group (all *p *> .05). In patients with BD, however, the model that included 2‐back working memory performance (*β* = −0.722) significantly predicted negative symptoms (*F*_(1,7)_ = 6.531, *p* = .043), explaining 44% of the variance of negative symptom severity scores. The same model (working memory *β* = −0.718) significantly predicted PANSS general scores (*F*_(1,7)_ = 6.370, *p* = .045), explaining 43% of the variance. No other model was predictive of psychotic symptoms (all *p *> .05).

### 3.5 Multivariate analyses for psychotic symptom severity differences between clinical group and early‐life stress occurrences

To examine differences in psychotic symptom severity between both clinical groups (SZ and BD) and ELS experience, reported ELS experience was dichotomized into absent levels (e.g., individuals who reported no ELS experience; SZ = 4, BD = 4) and present levels (e.g., individuals who reported at least one event of ELS, SZ = 20, BD = 13). A 2 x 2 design (SZ vs. BD and absent ELS vs. present ELS) MANOVA revealed no interaction effect between the clinical groups and the occurrence of ELS (Wilks' *λ* = .911, *F*_(4, 30)_ = 1.145, *p* = .344, partial *η* ^2^ = .089). While a significant main effect for clinical group was found (Wilks' *λ* = .794, *F*_(3, 35)_ = 3.021, *p* = .043, partial *η* ^2^ = .206), there was no significant group difference in severity of clinical symptoms for PANSS total scores, PANSS positive symptoms, PANSS negative symptoms, or PANSS general symptoms (all *p *> .05). There was also no main effect found for ELS experience (absent vs. present levels; Wilks' *λ* = .970, *F*_(3, 35)_ = 0.355, *p* = .786, partial *η* ^2^ = .030).

## 4 DISCUSSION

The main aims of this study were to examine the association between the occurrence of ELS and severity of psychotic symptoms in PSY. Furthermore, we studied whether glutamate concentrations in prefrontal brain regions would mediate this relationship between ELS experience and psychotic symptom severity.

One of the main findings was that patients with PSY experienced significantly higher occurrences of ELS than HC, which is consistent with previous research (Bailey et al., 2018; Varese et al., 2012). This finding of greater prevalence of occurrences of ELS in PSY compared to the general population is at the basis of gaining a better understanding of the etiology of psychotic symptoms following the experience of ELS (Barker, Gumley, Schwannauer, & Lawrie, 2015; Chase et al., 2019; Read, Fosse, Moskowitz, & Perry, 2014). The elucidation of this potential relationship has significant clinical and research implications for improving treatment avenues, such as pharmacological treatment (based on altered glutamate concentrations) or cognitive remediation trainings (based on altered working memory function). In our additional analyses focusing on both SZ and BP, we found that SZ reported higher ELS exposure when compared to HC, whereas BD showed comparable levels to HC. This latter finding contrasts previous meta‐analyses, which suggested that ELS occurrences were significantly higher among BD compared to HC (Etain et al., 2010; Macmillan et al., 2004). A possible reason for this discrepancy is the choice of ELS measure of the CLEQ in this study when compared to the Childhood Trauma Questionnaire (CTQ) among others (Dauvermann et al., 2017).

Contrary to our hypothesis, the occurrence of ELS was not significantly associated with the severity of psychotic symptoms in patients with PSY. This contrasts previous studies in which positive correlations between these two measures were revealed (Janssen et al., 2004; Kay et al., 1987; Öngür et al., 2008). One reason for this discrepancy could be the choice of ELS measure of the CLEQ this study. Unlike commonly used measures of childhood trauma or early‐life adversity, such as the CTQ (Dauvermann et al., 2017) or the Adverse Childhood Experiences (The ACE Score, 2003), the CLEQ does not directly ask participants whether they have experienced abuse (e.g., physical abuse) or neglect (e.g., emotional neglect) but provides an open‐ended question for such experiences to be reported (“Are there are any other significant life events you experienced as a child that are not mentioned above?”). This open question may have resulted in an under‐reporting of abusive and neglectful experiences in this study. Support for this interpretation comes from a previous meta‐analysis which reported that, dependent on adversity type, between 26% and 39% of participants with PSY reported experiencing trauma as a child (Bonoldi et al., 2013). However, this figure was just 12.2% for individuals with PSY in this study. Despite the established association between childhood trauma and severity of psychotic symptoms (Agnew‐Blais & Danese, 2016; Bailey et al., 2018; Bechdolf et al., 2010; Day et al., 1987; Gershon et al., 2013; Holtzman et al., 2013; Lataster et al., 2012; Mayo et al., 2017; Os et al., 2010; Shannon et al., 2011; Thompson et al., 2009; Üçok et al., 2015; Varese et al., 2012; Walder et al., 2014), it is also known that other environmental risk factors, such as cannabis use, migration and urbanicity are highly correlated with psychosis (Murray et al., 2017; Neilson et al., 2017; Newbury et al., 2018). Recently, it has been suggested that several measurements for the assessment of ELS should be used to cover different aspects of ELS (Vargas et al., 2019).

In the current study, ELS experiences were not significantly associated with working memory performance despite finding that individuals with PSY had significantly increased ELS experience and reduced working memory performance relative to HC. These group differences for both the low and high difficulty levels during working memory are consistent with meta‐analyses and systematic reviews repeatedly confirming this (Forbes, Carrick, McIntosh, & Lawrie, 2009; Frydecka et al., 2014; Hamilton et al., 2009). We speculate that the lack of a significant relationship between ELS and working memory in our study may be due to the working memory data utilized. In other words, it is possible that a neurobiological measure of working memory function in the form of blood‐oxygen‐level‐dependent (BOLD) response of brain activity as measured with functional magnetic resonance imaging (fMRI) may be required to reveal a potential mediation role of reduced working memory function on the relationship between ELS and severity of psychotic symptoms. Support for this interpretation comes from inconsistently reported relationships between ELS experience and working memory function across studies (Dauvermann & Donohoe, 2019a; Goodman et al., 2019; Vargas et al., 2019) when behavioral performance is considered. In contrast, widely established findings of altered BOLD responses of the DLPFC in individuals with PSY when compared to HC during working memory (Glahn et al., 2005; Owen, McMillan, Laird, & Bullmore, 2005) emphasize the greater reliability to study the role of working memory on the severity of symptoms, while the laterality of the DLPFC underlying working memory function seems to be variable across studies. In addition, a recent study linking glutamate concentrations as well as glutamate + glutamine (Glx) levels during a working memory task adds further support to the potential relevance of using brain function during working memory in individuals with psychosis (Jelen et al., 2019). Emerging evidence for utilizing brain activation data during working memory to study this association between ELS exposure and working memory also emphasizes the recruitment of brain activation of the DLPFC in HC with the experience of childhood trauma (Philip et al., 2013, 2016; Teicher & Samson, 2016; Teicher, Samson, Anderson, & Ohashi, 2016) and rodents following chronic stress exposure (Onaolapo et al., 2019; Shao et al., 2015; Yuen et al., 2011).

Focusing on the ^1^H‐MRS data, individuals with PSY demonstrated significantly reduced glutamate concentrations in the left DLPFC compared to HC. Previous research on differences in glutamatergic neurotransmission (e.g., glutamate, Glx and Gln concentrations) in BD, SZ, and HC has produced mixed findings. Studies have reported increased Glx concentrations in the left DLPFC in BD relative to HC (Frey et al., 2007; Michael et al., 2003; Michael, Erfurth, & Pfleiderer, 2009; Taylor, 2014), but significant differences in Glx between individuals with SZ (regardless of medication status) and HC have not been observed (Kaminski et al., 2020). Meta‐analytical research has reported inconsistent findings between studies regarding glutamate concentrations in the DLPFC (both right and left) in patients with SZ and HC. Furthermore, there were no significant differences in glutamate concentration in the right DLPFC or ACC in individuals with PSY compared to HC. Findings from previous studies in these areas are also inconsistent (Marsman et al., 2013; Merritt, Egerton, Kempton, Taylor, & McGuire, 2016; Poels et al., 2014). However, a number of studies have found no evidence of group differences in glutamate, glutamine, or Glx concentrations between chronic medicated patients with SZ and HC (Bustillo et al., 2011; Kraguljac et al., 2012; Reid et al., 2010; Rowland et al., 2013; Shirayama et al., 2010 *).* A limitation of the previous research is that many studies interpret glutamate, Gln, and Glx concentrations interchangeably, so BD‐ and SZ‐related increases (Gigante et al., 2012; Théberge et al., 2003) that are specific to one of these indicators (e.g., Gln or Glx) but not others may complicate the picture. Further research is required to better understand the roles of each of the glutamate‐related metabolites in the DLPFC and ACC in PSY.

Finally, our novel findings of this study are that ELS occurrences were inversely associated with glutamate concentrations in the left DLPFC for the HC group only. Previous studies on HC focused on the effect of acute stress on the PFC, with reports of both elevated Glx concentrations (Zwanzger et al., 2013) and no change in glutamate levels (Houtepen et al., 2017). To our knowledge, this is the first study providing preliminary evidence of a link between ELS exposure and altered glutamate concentrations in the DLPFC in humans, which is supporting impairments of glutamatergic neurotransmission previously reported in rodents after chronic social stress (Aas et al., 2012; Janssen et al., 2004). However, we did not observe a significant relationship between ELS experience and glutamate concentrations in individuals with PSY. This observed association in HC only is comparable to a stronger relationship between the experience of ELS and deficits in neurocognitive function (including working memory) in HC than in individuals with PSY (Malarbi, Abu‐Rayya, Muscara, & Stargatt, 2017; Philip et al., 2016; Vargas et al., 2019). It has been suggested that such a finding could be due to the fact that the effect of ELS cannot be isolated from other known implicated factors on cognitive function and severity of psychotic symptoms in PSY, such as genetic risk factors, current and acute stress levels (Dauvermann & Donohoe, 2019b), and medication effects (Dauvermann & Donohoe, 2019a; Vargas et al., 2019). However, given the strong link between ELS and increased likelihood of developing PSY and the support of aberrant glutamatergic transmission in the DLPFC in PSY is it also possible that the lack of significant findings in individuals with PSY was limited by methodological shortcomings, including sample size.

Future research studying relationships between ELS, cognitive function, and neurobiological markers in PSY and other stress‐sensitive psychiatric disorders should focus on overcoming limitations of a variety of neurocognitive batteries and ELS measures (Dauvermann & Donohoe, 2019a; Vargas et al., 2019) that are challenging to interpret. Emerging evidence on combining ELS, cognitive, endocrine, cytokine, and neuroimaging data will lead to greater insight into the interrelationships and therefore etiology of PSY.

## CONFLICT OF INTEREST

The author SML has received financial support for research, in the past 3 years, from Janssen, in relation to research. He has also received fees for advisory panels and/or educational meetings from Janssen and Sunovion. HCW is supported by a JMAS SIM Fellowship from the Royal College of Physicians of Edinburgh and by an ESAT College Fellowship from the University of Edinburgh. SML and HCW have also previously received support from Pfizer (formerly Wyeth). These received funds do not present a conflict of interest with the present study.

## AUTHOR CONTRIBUTIONS

D.O.H. and M.R.D. conceived the idea of the presented work. M.C. and E.L.H. analyzed the data. M.C. wrote the manuscript with support from D.O.H. and M.R.D. H.C.W., J.H., and S.M.L. conceived the idea of the original work. All authors discussed the results and contributed to the final manuscript.

## Supporting information

Supplementary Material

## Data Availability

The data that support the findings of this study are available from the corresponding author upon reasonable request.
