# Supplementary material for: Are working memory and glutamate concentrations involved in early‐life stress and severity of psychosis?
Source: Brain Behav. 2020 May 9;10(6):e01616. doi: 10.1002/brb3.1616 (PMC7303391; doi:10.1002/brb3.1616)
Supplement: Supplementary file 1 — Supplementary Material [file BRB3-10-e01616-s001.docx]

**Title: Do working memory, glutamate concentrations and early life stress play a role in the severity of psychosis?**

**Running Title**: Factors in Early-life stress and psychosis severity

**Authors**: Mark Corcoran^a^, Emma L. Hawkins^b^, Denis O’Hora^a^, Heather C. Whalley^b^, Jeremy Hall^c^, Stephen M. Lawrie^b^, Maria R. Dauvermann^a*^

**Institutional Affiliations**:

^a^ School of Psychology, National University of Ireland Galway, University Road, Galway, Ireland

^b^ Division of Psychiatry, University of Edinburgh, Edinburgh, UK

^c^ Neuroscience and Mental Health Research Institute, Cardiff University School of Medicine, Cardiff, UK

^d^ McGovern Institute for Brain Research, Massachusetts Institute of Technology, Cambridge, USA

^e^ Department of Psychiatry, University of Cambridge, Cambridge, UK

*Corresponding author:

Dr Maria Dauvermann, PhD

Postdoctoral Researcher

School of Psychology

National University of Ireland Galway

maria.dauvermann@nuigalway.ie

Tel: +353 91495953

**Appendix S1 Supplementary Methods**

**Experimental details and processing - N-back working memory task**

The N-back task parametrically increases the demands placed on working memory (WM), in this case over three levels (0-back, 1-back and 2-back). It was implemented as a blocked design, with letter stimuli being presented every 3s for 1s each, 14 per block. Blocks were preceded by a 7.5s instruction period, which informed the participant if this was to be a 0-, 1- or 2-back period. Total block duration was 49.5s. There were 3 repetitions of each level. Participants were asked to press button A if they saw a target letter, and B for any other. In the 0-back condition, the target was simply the letter X. For 1-back, the target was any letter that was the same as the immediately preceding one, and for 2-back the target was any letter that was the same as the one before that.

**Magnetic Resonance Spectroscopy data acquisition and analysis**

**MRS protocol**

The voxel placement followed a standardized protocol, with navigation steps to determine the coronal slice for placement. Cortical feature identification was used to designate the voxel centre, followed by rotations of the voxel in the transverse and sagittal views to obtain the final placement.

The location of the ACC voxel is shown in Figure 1A. The coronal slice for voxel placement was found by moving 15 mm posterior from the genu of the corpus callosum (CC). In this coronal view the centre of the voxel was placed on the mid-line of the inter-hemisphere fissure, and 15mm above the dorsal surface of the CC. In the transverse view the voxel was rotated so that the anterior-posterior (long) axis followed the midline of the inter-hemisphere fissure. In the sagittal slice, the voxel anterior surface was aligned with the anterior edge of the genu of the CC. The voxel long axis was then rotated to follow the contour of the CC.

The placement of the left DLPFC voxel is shown in Figure 1B. The Talairach coordinates of the centre of the DLPFC voxel were x = +/-26, y = +24, z = +34. The coronal slice for voxel placement was found by moving 10 mm posterior from the genu of the corpus callosum. Then the voxel was centred in the white matter bordering the grey matter of the middle frontal gyrus (MFG). The transverse and sagittal slices were set by repeating the centering of the voxel in the white matter bordering the grey matter of the MFG. The voxel was then rotated to follow the contour of the cortex in the sagittal plane.

**Results**

Table S1. Demographic and clinical details for healthy controls and clinical subgroups

|  | All | HC | Psychosis | | Test | *p* ^¶^ |
| --- | --- | --- | --- | --- | --- | --- |
|  |  |  | SZ | BD |  |  |
| N | 88 | 41 | 30 | 17 |  |  |
| Age Mean (SD) | 39.5 (12.99) | 38.29 (14.44) | 38.33 (10.47) | 44.64 (12.77) | *F* | .197 |
| Sex (M:F) | 63:32 | 23:18 | 22:8 | 11:16 | χ *^2^* | .543 |
| Education (1:2:3) ^†^ | 18:9:48 | 10:2:24 | 5:5:14 | 3:2:10 | χ *^2^* | .928 |
| PANSS Total ^‡^ Mean (SD) | 13.23 (17.11) | - | 25.13 (19.02) | 20.00 (15.62) | *t* | .220 |
| PANSS Positive ^‡^ Mean (SD) | 2.91 (4.48) | - | 6.53 (5.41) | 3.12 (3.30) | *t* | .046* |
| PANSS Negative ^‡^ Mean (SD) | 3.55 (6.08) | - | 7.36 (7.71) | 4.65 (5.71) | *t* | .160 |
| PANSS General ^‡^ Mean (SD) | 6.78 (8.40) | - | 11.23 (8.46) | 12.24 (9.04) | *t* | .543 |
| CLEQ ^§^ Mean (SD) | 1.88 (1.81) | 1.23 (1.48) | 2.83 (1.95) | 2.00 (1.77) | *F* | .002* |
| Antipsychotic medication (Yes:No) | - | - | 15:7 | 5:1 | - | - |
| Mood Stabiliser medication (Yes:No) | - | - | 8:14 | 2:4 | *-* | - |
| Antidepressant medication (Yes:No) | - | - | 6:16 | 1:5 | *-* | - |

HC; Healthy Controls, SZ; Schizophrenia, BD; Bipolar Affective Disorder, PANSS; Positive and Negative Symptom Scale, CLEQ; Childhood Life Events Questionnaire

^†^ 0, Compulsory; 1, More than compulsory; 2, Post-Secondary

^‡^ Rescaled total PANSS scores

^§^ Rescaled CLEQ scores

^d^ Two-tailed test

* *p* < .05, ** *p* < .001

Table S2. Glutamate concentrations in institutional units (IU) ^‡^

|  | HC | SZ | BD | Test | *p* |
| --- | --- | --- | --- | --- | --- |
| R DLPFC | 8.64 (2.11)  *n* = 33 | 8.31 (1.45)  *n* = 18 | 7.59 (1.41)  *n* = 11 | *F* | .464 |
| L DLPFC | 8.20 (1.36)  *n* = 34 | 7.36 (1.18)  *n* = 20 | 7.48 (1.60)  *n* = 13 | *F* | .062 |
| ACC | 7.79 (1.71)  *n* = 28 | 7.38 (1.81)  *n* =20 | 6.90 (1.99)  *n* =15 | *F* | .846 |

ACC, Anterior Cingulate Cortex; BD, individuals with bipolar disorder; HC, Healthy Controls; L DLPFC, Left Dorsolateral Prefrontal Cortex; R DLPFC, Right Dorsolateral Prefrontal Cortex; SZ, individuals with schizophrenia.

* *p* < .05

^†^ Participants with Glutamate concentration standard deviation exceeding 15% were excluded from this table.

^‡^ Age and sex were entered as covariates
